# *EXEQ* and *InEXEQ*: software tools for experiment planning at the Extreme Environment Diffractometer

**DOI:** 10.1107/S1600576720011942

**Published:** 2020-10-19

**Authors:** Maciej Bartkowiak, Karel Prokeš, Michael Fromme, Anne Budack, Juliane Dirlick, Oleksandr Prokhnenko

**Affiliations:** a Helmholtz-Zentrum Berlin für Materialien und Energie GmbH, Lise Meitner Campus, Hahn-Meitner-Platz 1, 14109 Berlin, Germany

**Keywords:** software, neutron diffraction, inelastic neutron scattering, experiment planning

## Abstract

The Extreme Environment Diffractometer was a neutron time-of-flight instrument equipped with a constant-field hybrid magnet providing magnetic fields up to 26 T. The magnet infrastructure and sample environment imposed limitations on the geometry of the experiment, making it necessary to plan the experiment with care. *EXEQ* is the software tool developed to allow users of the instrument to find the optimal sample orientation for their diffraction experiment. *InEXEQ* fulfilled the same role for the inelastic neutron scattering experiments. The source code of the software is licensed under the GNU General Public Licence 3, allowing it to be used by other facilities and adapted for use on other instruments.

## Abstract 2

*EXEQ*/*InEXEQ* software is a dedicated tool for single-crystal orientation at the HFM-EXED neutron scattering facility, and also a mandatory step of the proposal submission process.

## 1. Introduction   

The High Field Magnet (HFM)  (Smeibidl *et al.*, 2016 ▸) and the Extreme Environment Diffractometer (EXED)  (Prokhnenko *et al.*, 2015 ▸) were the two mutually optimized components of the HFM-EXED facility at the BER-II reactor at HZB Berlin. At the time of writing this paper, BER-II has been shut down and its decommissioning is already in progress. While still in operation, HFM-EXED had been the only neutron instrument in the world working with constant magnetic fields up to 26 T. This publication describes the design and functionality of the *EXEQ Calculator* (EXED E,Q-range calculator), which was a software tool written for the EXED instrument to calculate the instrument coverage, verify experimental plans of proposals and assist the identification of Bragg reflections observed in the experiment.

EXED is a neutron time-of-flight (TOF) instrument. Originally it operated in diffraction mode and a low-*Q* mode; an additional upgrade made it possible to perform inelastic neutron scattering (INS) experiments on EXED  (Bartkowiak *et al.*, 2015 ▸). The HFM is a horizontal magnet with a cylindrical 50 mm warm bore in the centre of the magnet and two 30°-wide conical openings aligned coaxially with the bore. The magnet could be rotated around the vertical axis in the range of −11.85 to +2.4°. During the magnet rotation the forward detector panels moved continuously with the magnet, while the backward panels remained fixed in place. Out of the three cryostats available on the instrument, in the ^4^He cryostat it was possible to rotate the sample around the vertical axis, and in the remaining two cryostats (a conventional ^3^He cryostat and a dilution refrigerator) no sample rotation was possible. At the same time, the cooling time needed to reach the base temperature when starting from ambient conditions ranged from 12 to 24 h. This means that not only was it impossible to change the sample orientation inside the cryostat, but it was also not possible to accommodate an unplanned sample orientation change *ex situ* within the allocated experiment time. The only other method of adjusting the instrument coverage was to rotate the entire magnet around the vertical axis, effectively rotating the sample relative to the incoming neutron beam, while maintaining the sample orientation relative to the field. The limitations listed in this paragraph made it essential for the success of each experiment to find the optimal sample orientation well in advance.

Given the unusual configuration of the instrument, it was not possible to take advantage of the existing software for sample orientation. The common assumption has been that movable instrument parts are not present on neutron TOF instruments. Software such as *Horace Planner* (Ewings *et al.*, 2016 ▸) is designed to calculate the coverage of neutron TOF spectrometers. However, it does not support instruments in which the detector positions change during the measurement. Software such as *CrystalPlan* (Zikovsky *et al.*, 2011 ▸) can optimize the sample orientation on a diffractometer in order to maximize the percentage coverage of the reciprocal space. In the case of the HFM-EXED facility, the coverage is limited to the extent that only a small number of Bragg peaks can be observed, and instead of the statistical approach it is necessary to choose a specific set of peaks and bring them into the covered range.

The reasons outlined above made it necessary to develop *EXEQ* for the planning of the diffraction experiments at the HFM-EXED facility. Later on, as EXED had been upgraded to operate also as a direct TOF spectrometer, *InEXEQ* was developed from the existing code base of *EXEQ*. Both programs have been designed specifically to match the mode of operation of the EXED instrument and help users of the instrument overcome the limitations of EXED’s scattering geometry. Performing a calculation with either *EXEQ* or *InEXEQ* was a mandatory step of experimental proposal submission at the HFM-EXED facility throughout its entire operation.

Note that the *Mantid* package (Arnold *et al.*, 2014 ▸) contains the *DGSPlanner* interface, and starting from *Mantid 3.10* it can also be used to calculate the covered range of the EXED instrument in the inelastic mode. The results produced with *DGSPlanner* are equivalent to those produced by *InEXEQ*. However, *InEXEQ* has the advantage of being able to calculate the chopper speeds and neutron flux of EXED for the given value of requested energy resolution, allowing users to optimize the chopper settings easily. Also, both *EXEQ* and *InEXEQ* can produce plots of the detector panels, and map the selected points in the reciprocal space onto the detectors, allowing users to anticipate problems caused by the sample signal appearing too close to the edge of the detector or to the shadowed parts of the detector.

## 2. Software dependencies and availabiliy   

Since all users of the HFM-EXED facility were required to use either *EXEQ* or *InEXEQ*, an essential criterion when designing the software was not to limit the user base by relying on a restrictive or expensive licence; for this reason, open-source solutions were chosen for all the components of the software. The main part of the code was written in Python, using the *NumPy* (van der Walt *et al.*, 2011 ▸) and *SciPy* (Virtanen *et al.*, 2020 ▸) modules for the computational part, *PyYAML* (https://pyyaml.org/) for reading and writing of parameter files, and *Matplotlib* (Hunter, 2007 ▸) for creating the plots and coverage maps. The original graphical user interface (GUI) was based on *Tk* (http://www.tcl-lang.org/) using the *Tkinter* module (https://wiki.python.org/moin/TkInter). The binary builds of the software, available for download, were created using *PyInstaller* (http://www.pyinstaller.org/).

As support for Python 2 expired at the beginning of 2020, the final update of the software has been released, making the code compatible with Python 3, and replacing the old GUI with a new one, based on *PyQt5* (https://pypi.org/project/PyQt5/).

Both *EXEQ* and *InEXEQ* are free software, released under the GNU General Public License 3, allowing users to read, modify and re-distribute the code under a compatible licence. Information and downloads for the *EXEQ*/*InEXEQ* software are available at the instrument website: https://www.helmholtz-berlin.de/pubbin/igama_output?modus=einzel&gid=1939.

## 3. Methods   

### 3.1. Instrument   

The definition of the instrument includes the detector positions, the cryostat wall positions and the neutron wavelength spectrum. When *EXEQ* was being written, the EXED instrument used several fixed, pre-defined detector positions for measurements, while the magnet rotated continuously, additionally shadowing the detector panels. Therefore, the instrument definition was loaded from XML files and cut-offs were calculated for each detector tube to determine the total solid angle covered by the detectors. Later on, a vacuum chamber for the INS mode was installed with rotatable detector panels larger than the magnet cone, allowing for the calculation to be simplified in *InEXEQ*, where the entire forward opening of the magnet cone is covered, excluding the beamstop position and the gaps between panels. This simplification of the detector definition to basic geometrical shapes allowed us to implement all the processing as *NumPy* functions, resulting in a significant speedup over the original *EXEQ* code, which implemented the tests of detector limits in pure Python. However, *EXEQ* still uses *Mantid*-style XML files for storing the instrument definition, and replacing the EXED instrument with another one from the *Mantid* instrument directory should not require a significant programming effort.

The calculation of the flux is based on the simulation of the instrument performed using *VITESS*  (Wechsler *et al.*, 2000 ▸; Zsigmond *et al.*, 2002 ▸), where the neutron spectrum at the sample position was simulated for the entire neutron guide system, neglecting the choppers. These results are interpolated using the *SciPy* interpolation module, and then scaled by a reduction factor calculated from the chopper settings.

### 3.2. Sample   

The sample definition is common to both *EXEQ* and *InEXEQ*. The unit cell is defined by three lattice constants *a*, *b* and *c*, and three angles α, β and γ. The space group of the sample is not a part of the input, nor are the atomic positions inside the unit cell. Therefore, symmetry considerations do not enter the calculations in the software, and it is up to the user to choose a valid position in the reciprocal space. However, this way the input is also not limited in any way, and it is just as easy to specify a reflection corresponding to an incommensurate structure as it is to specify a Bragg reflection with integral Miller indices.

The sample orientation is defined using the original definition  (Busing & Levy, 1967 ▸) of the *UB* matrix. However, users are expected to use 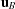 and 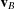 vectors (labelled Bu and Bv, respectively, in the software interface) instead of the usual 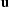 and 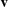. The 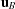 and 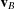 vectors define the sample orientation with respect to the magnetic field of the instrument and not to the neutron beam; they are identical to 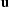 and 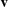 when the magnet rotation angle is 0°, and are independent of the magnet rotation.

**Figure 1:**
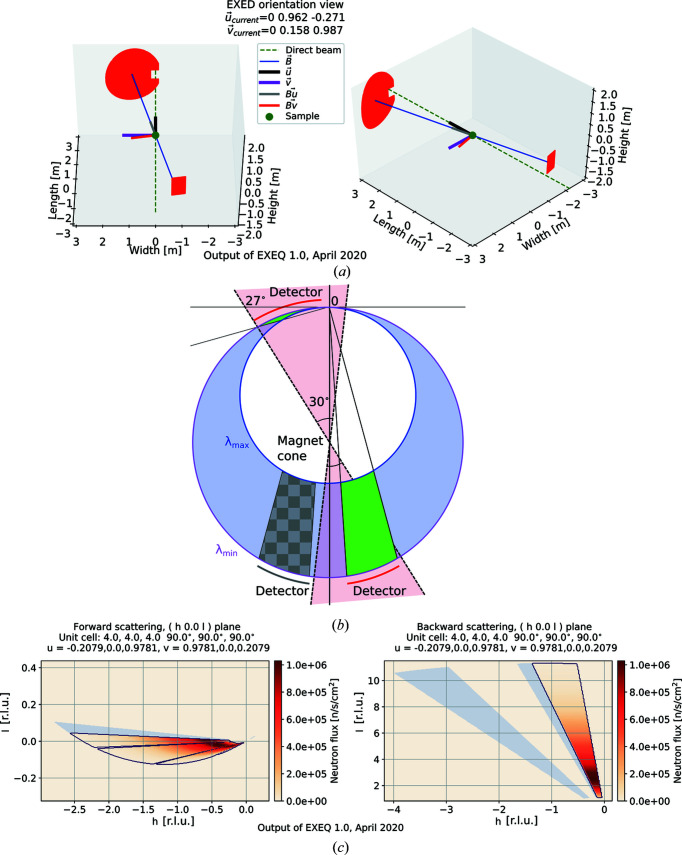
(*a*) Sample orientation view (in real space), common to both *EXEQ* and *InEXEQ*. The sample is in the centre and the red shapes away from the sample indicate the usable area of the detector. The conventional 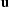 and 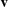 are shown together with the 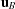 and 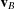 (labelled Bu and Bv, respectively) used as user input. The configuration shown here corresponds to the largest possible angle of magnet rotation, and to the maximum instrument coverage. (*b*) The Ewald construction showing the coverage of the instrument corresponding to the detector positions shown above. The dashed lines indicate the magnet cone limits (in real space), affecting the accessible detector area, and the circles indicate the limits of the wavelength band. The covered range (in reciprocal space) is marked in green, and the range blocked by the magnet cone in checkered grey. (*c*) The reciprocal-space coverage calculated with *EXEQ* for a cubic sample using the orientation shown above. The grey area outside the contour indicates the shadowed part of the detector.

Simple utility functions of both *EXEQ* and *InEXEQ* include plotting the sample orientation. The detector positions, beam direction and magnetic field direction are plotted in real space, and the directions of 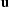, 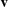, 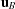 and 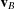 are indicated in the same coordinate system. This should remove any ambiguity related to the convention of axis labelling or handedness of the coordinate system, as the effect of all the goniometer rotations is shown in real space. Fig. 1 ▸(*a*) shows an example of the sample orientation plot, generated for −12° magnet rotation angle. (The extreme position of the detector chamber in the real instrument is reached at −11.85°, but −12° is still allowed in the calculator to simplify the input.) At this position, the edge of the magnet cone is located 27° away from the direction of the direct beam. The red areas in Fig. 1 ▸(*a*) indicate the positions of accessible detector panels in real space; as this is a real-space plot, it is independent of the definition of the sample unit cell. For this configuration, the Ewald construction is shown in Fig. 1 ▸(*b*). The accessible volume is contained between the two Ewald spheres, corresponding to the largest and smallest neutron wavelengths of the current wavelength band; then, the accessible volume is cut by the angular coverage of the detectors. The combination of these conditions gives the covered volume, marked in green in the figure. The dashed lines indicate the geometrical limits and accessible detector surface in real space, and the green areas indicate the coverage in reciprocal space; therefore, the fact that the dashed lines intersect with the green area is of no significance to the calculation, since the two belong to different coordinate systems. Fig. 1 ▸(*c*) shows the reciprocal-space coverage calculated using *EXEQ* for the same instrument configuration assuming a cubic sample with the lattice constant *a* = 4 Å, which is the default sample definition in *EXEQ*. Since the sample cryostat is rotated together with the magnet, the −12° magnet rotation results not only in the change of detector position [already shown in Figs. 1 ▸(*a*) and 1 ▸(*b*)] but also in a rotation of the sample. Therefore, a −12° rotation is applied to the sample, and the final **u** and **v** orientation vectors with respect to the incoming beam are **u** = (−0.2079, 0, 0.9781) and **v** = (0.9781, 0, 0.2079) instead of the original 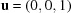 and 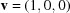 at 0° magnet rotation. This way the coverage in the *hkl* coordinates shown in Fig. 1 ▸(*c*) is rotated compared with the ideal calculation in Fig. 1 ▸(*b*). The grey areas in the plot indicate the shadowed part of the detector panels. This means that a detector pixel was present at the real-space position corresponding to the reciprocal-space coordinates in the plot, but the flight path between the sample and that pixel was blocked. This is in contrast to the other points in the colour map marked as having zero neutron flux, which correspond to a position in real space where no detector is present or no neutrons of appropriate wavelength are present in the instrument wavelength spectrum.

In both the *EXEQ* and *InEXEQ* codes, the main part of the calculation relies on the inverse *UB* matrix to transform the *hkl* coordinates into the scattering vector 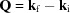, where 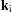 and 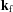 indicate the initial and final neutron wavevectors, respectively. Then, depending on which software is being used, the 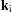 and 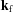 vectors are derived from 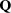 assuming either elastic scattering (where 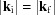) in the case of *EXEQ* or inelastic scattering (where 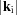 is known from the instrument settings) in the case of *InEXEQ*. The coordinate system is chosen so that the *z* axis coincides with the incoming neutron beam, the *x* axis is perpendicular to the beam in the horizontal plane and the *y* axis is vertical. This way 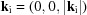 in both the elastic and the inelastic scattering mode.

The equations used in the calculation are well known and standard in the neutron scattering community: the *UB* matrix (or, more precisely, the *RUB* matrix, where *R* contains the rotations resulting from the goniometer angles) defines the relationship between the 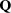 vectors and the *hkl* coordinates: 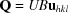 and 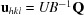, where 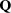 is the scattering vector in the instrument frame, and 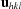 is the reciprocal-space vector expressed in the basis of the reciprocal-lattice vectors 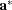, 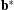 and 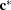. The advantage of using the *EXEQ*/*InEXEQ* software lies in the fact that the behaviour of the instrument is reproduced exactly in the calculation. This means that the rotation of the magnet results automatically in an identical rotation of the sample. Also, the maximum rotation angles of the magnet are adjusted depending on the choice of the last segment of the neutron optics (where the choice is currently between a neutron guide and a collimation system for low-*Q* experiments).

### 3.3. *EXEQ*   

The required input, apart from the sample orientation, is the wavelength range 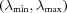 and the requested resolution at the band centre 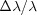. The simulated spectrum of the instrument is used in order to calculate the flux at each wavelength, and the resolution is the input for the chopper frequency calculation, which determines the flux reduction factor. This way a realistic prediction can be made for the flux in the experiment.

**Figure 2:**
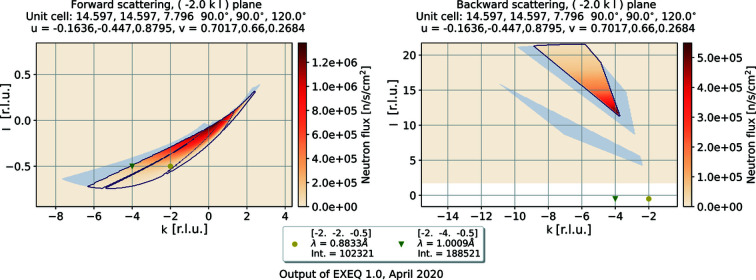
A map of the instrument coverage generated using *EXEQ*. The left (right) side shows the forward (backward) detector panels. Inside the contour, the colour map corresponds to the expected neutron flux at each point, where a darker colour indicates higher flux. The grey area outside the contour indicates the shadowed part of the detector. The points of interest selected by the user are marked in both parts of the map.

In *EXEQ*, the calculation of the coverage starts with the user input defining the plane to be plotted. At the moment, the possibility of plotting arbitrary planes has not been added to the interface; therefore the plane has to be orthogonal to the *h*, *k* or *l* direction, keeping the other two directions in plane. A simple example of the coverage map has already been shown in Fig. 1 ▸. In Fig. 2 ▸, the calculation has been performed using the unit-cell definition of green dioptase, a naturally occurring mineral. The coverage is plotted for 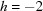, since the reflections of interest are located in this plane. The requested reflections are marked on the map, and their corresponding neutron wavelength and flux are shown in the legend of the plot. The reflections of interest are marked in both parts of the plot, to allow users to note easily if their reflection of interest could be reached using the backscattering panels rather than the forward panels.

**Figure 3:**
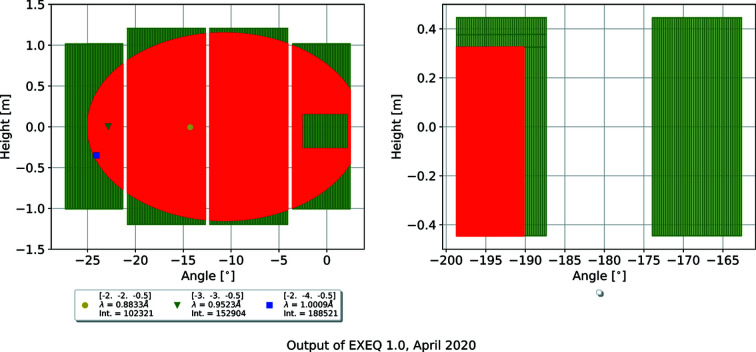
Detector view of *EXEQ*. The detector position matches the selected magnet rotation angle. The specified *hkl* points are marked on the detector in real space. The shadows of the magnet cone, chopper housing and beamstop are marked in green, while the remaining accessible area is solid red.

Another output of *EXEQ* is the plot of detector panel positions, shown in Fig. 3 ▸. The real-space positions of the reflections are marked on the detector. (The set of input parameters used here is the same as for the coverage map in Fig. 2 ▸.) There is no calculation of the total size of the peak on the detector; the calculation shows only the ideal centre of the peak.

Internally, the calculation is performed in several steps. First, the limits of the coverage are approximated by calculating the 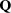 vector for a sparse set of pixels distributed over the edge of the detector array at 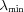 and 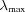. Then, a rectangular grid of *hkl* points is created within these limits, and 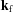 is calculated for each point on the grid. For a vector 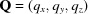 the scattering vectors can be found using the relationship 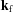 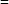 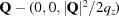. The length of the vector, 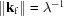, and the direction are given as two angles in a spherical coordinate system. For each point, if the angles fall within the limits of a detector panel (including shadowing by the beamstop and the cryostat walls) and the wavelength is within the chosen limits, the point is given the value of the expected neutron flux at this wavelength; the value is 0 otherwise.

**Figure 4:**
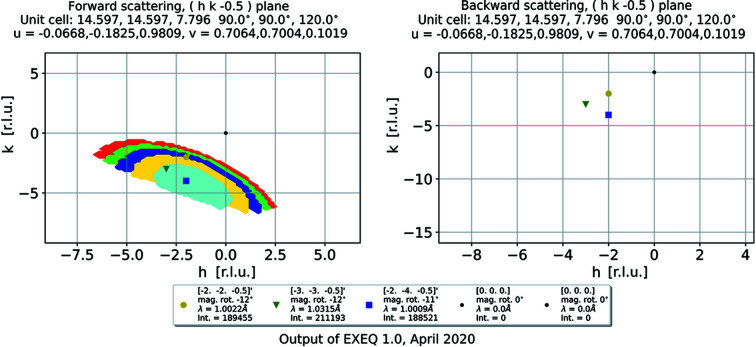
A map of the total instrument coverage generated using *EXEQ*. Every magnet rotation angle is plotted using a different solid colour. For each marked point, the legend shows the angle where the flux at that point is the highest. In this example, the backscattering panels do not cover any area of the requested plane.

A total coverage map (Fig. 4 ▸) can be generated, where the coverage plots calculated for different magnet rotation angles are superimposed. The map is meant to produce a qualitative result, informing the user if there is any possibility at all of accessing the part of the reciprocal space they requested. In order to obtain more detailed information it is necessary to specify a reflection of interest as an input parameter. For the reflections of interest defined by the user, which are marked in the plot, the magnet rotation angle resulting in the highest flux will be indicated for each marker. In Fig. 4 ▸, the requested value of 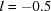 introduces an out-of-plane component to the scattering vector; as a result, the backscattering panels do not provide any coverage, independent of the magnet rotation angle.

### 3.4. *InEXEQ*   

The input for *InEXEQ* consists of the incoming neutron energy *E*_i_ in meV units and the required percentage resolution at the elastic line position. On the basis of these parameters, the chopper frequency is calculated, assuming a 1:1 ratio between the first and last chopper discs. Large chopper windows are used whenever possible, to maximize the incoming flux. The chopper frequency is also used in the calculation of the maximum energy transfer. The energy transfer range may be limited, as the very slow neutrons corresponding to the largest energy transfer may be lost due to frame overlap. Therefore, we do not consider neutrons with sample-to-detector flight time longer than the chopper period. The formulae describing the energy resolution are the same as in the original paper by Lechner (1991 ▸).

**Figure 5:**
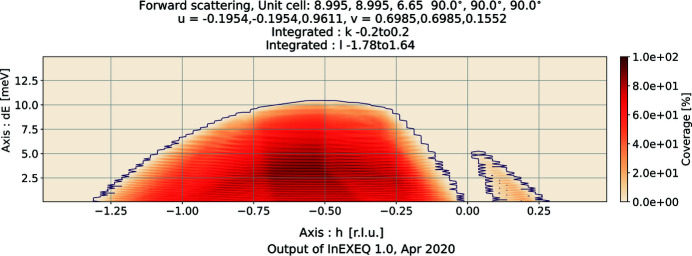
An example of a two-dimensional coverage map generated by *InEXEQ*. The gap in the coverage around *h* = 0 corresponds to the position of the beamstop.

**Figure 6:**
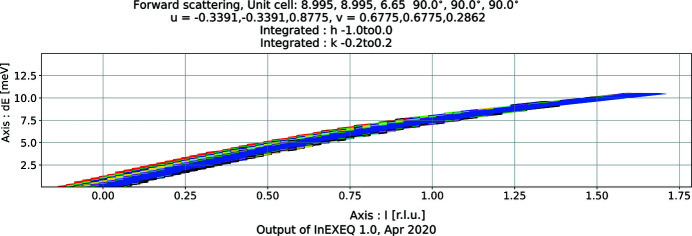
An example of a two-dimensional total coverage map generated by *InEXEQ*. Each colour corresponds to a different magnet rotation angle, in steps of 2°, resulting in eight rotation angles in total.

As opposed to *EXEQ*, the calculation here is performed on a four-dimensional grid of points. The points of the grid are evenly spaced in *hkl* and Δ*E* units. The range of the calculation can be given explicitly by the user or left for the software to find automatically. The main output of the software is the coverage map, where a one-, two- or three-dimensional plot is created, showing which points in the specified ranges of *hkl* and Δ*E* can be reached using the current input parameters. In the directions which are not used as plotting axes, the coverage is integrated. The ratio of the covered voxels to the total number of integrated voxels in each point is the percentage of coverage, which is shown as the intensity in the maps. An example of a single map is shown in Fig. 5 ▸ and a total coverage map is shown in Fig. 6 ▸. Just as was the case for the elastic scattering, the total coverage map is meant to be a coarse testing tool, telling users if the current sample orientation may provide access to the requested part of reciprocal space. As seen in Fig. 6 ▸, the coverage along the direction of the incoming neutron beam is nearly independent of the magnet rotation. The magnet rotation translates the detector array in the direction perpendicular to the plot plane, and rotating the crystal *l* direction with respect to the direct beam can change the momentum transfer along that direction only by 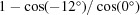 < 2.3%. Therefore, within the allowed rotation range there is always a part of the detector array at the angle corresponding to the reciprocal-space cut in the plot; at the same time, the sample orientation change is minimal. This example emphasizes the importance of precise experiment planning on the EXED instrument, as there is little room for correction once the sample is already in place.

Just as in *EXEQ*, the main part of the calculation consists of finding the 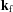 vector for each 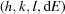 point of the grid. However, the conversion from 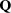 to 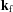 is simple in this case, since 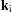 is 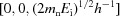, where *h* is the Planck constant, 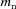 is the neutron mass and 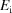 is the incident neutron energy which is known from the instrument settings.

The grid is generated as a *NumPy* array of 32-bit float variables, with the starting size of 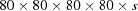, where *s* is the scaling factor labelled as ‘precision’ in the interface. This means that approximately 132 MB of RAM are necessary to perform the calculation with the standard precision of reciprocal-space sampling. If the user input requires fewer sampling points along any direction, the total size of the grid is preserved and the number of points along other axes is increased accordingly. Of course, there is still a risk that, by setting an unrealistically large range of coordinates along any axis, the user can produce a grid where none of the points coincide with the covered region. This is easily avoided by using the built-in automatic limit detection in the first step, and then narrowing down the coverage to the region of interest in order to increase the plotting precision further.

**Figure 7:**
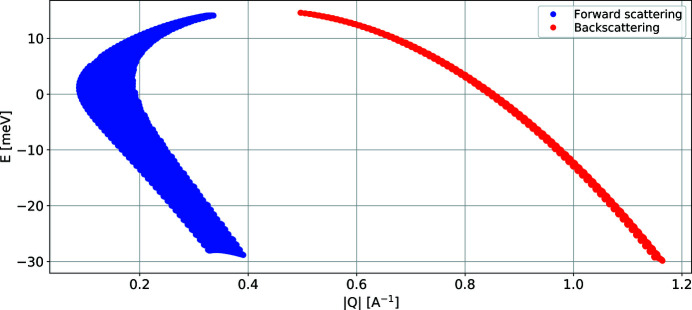
A plot of the maximum coverage for a powder sample, generated for *E*_i_ = 15 meV.

The maximum (*E*, *Q*) range of the instrument can be calculated, as shown in Fig. 7 ▸. This is mainly relevant to powder samples and is calculated assuming the maximum magnet rotation angle.

## 4. Interface   

*EXEQ* and *InEXEQ* have a modular structure, so that different parts of the code can be replaced without extensive changes to the remaining code. For this reason, it has been easy to implement different types of interfaces to the main part of the code. The standalone version offered a GUI; additionally, it was possible to run the code from the command line, using a text input file to pass the parameters to the code. The plots were then produced in *Matplotlib* using the *Agg* plugin. At the same time, the IT department of the Helmholtz Zentrum Berlin provided a web interface which would pass the input parameters to a server process running in the background and return the requested plots as a result.

The main design strategy for the interface was to provide the user with a set of reasonable default values, while at the same time allowing most of the parameters to be modified when needed. This is best seen in the case of *InEXEQ*, where it is enough to generate a coverage plot in order to obtain the coverage limits along every axis. Then, in the second iteration, the user can use these limits explicitly, and decide which axes should be used for plotting on a grid and which should be integrated.

Due to the computationally expensive initialization of the detector definition in *EXEQ*, the web interface required, on average, more time per calculation than the standalone version. However, it had the advantage of being accessible from any computer equipped with a web browser.

Both the standalone and web-based versions of the software were able to save the input parameters as text files and load them later, so that sample definition could be transferred from *EXEQ* to *InEXEQ*, or so that the instrument scientist could easily reproduce the calculation performed by users and suggest possible changes. To provide readers with an example, the supporting information with this paper includes the input files needed to recreate the plots shown in the figures.

## 5. Software applications   

The typical usage cycle of the software for each experiment progressed in several stages. Firstly, the proposers would calculate the coverage for the ideally aligned sample and verify if the features of interest (*e.g.* Bragg reflections, dispersion curves *etc*.) were accessible. The total coverage option could be employed here to find the magnet rotation angle for each peak at which the neutron flux at the peak position is the highest. If the desired features remained outside of the covered range, an intentional misalignment of the sample relative to the field could be introduced to improve the coverage, at the expense of the maximum magnetic field along the chosen crystallographic direction.

Secondly, the software was used to verify the sample orientation during the experiment. This was easily done by comparing the Bragg peak positions on the detector in the experiment with those calculated by the software. By adjusting the goniometer (misalignment) angles, it was possible to find the best match between the calculation and the measured data. This was essential for checking if the sample orientation relative to the magnetic field was within the expected tolerance.

Lastly, the sample definition and orientation could be transferred between *EXEQ* and *InEXEQ*, so that the INS coverage could be calculated for the real sample orientation. Using the sample orientation determined with *EXEQ*, it was possible to re-calculate the coverage and find the set of parameters that can compensate for a possible misalignment. This would typically involve rotating the magnet or changing the incoming neutron energy.

## 6. Conclusions   

The *EXEQ*/*InEXEQ* software was optimized to support the user programme at the HFM-EXED neutron scattering facility. The unique features of the instrument, such as the wide wavelength spectrum range, limited angular coverage and movable detectors in the time-of-flight mode, made it necessary to develop a new software package rather than use one of the already existing ones. The software was successfully applied throughout the entire user operation of HFM-EXED, and can still be used as an aid for interpretation of already measured data. Instruments built around horizontal magnets have been proposed in the past [*e.g.* ZEEMANS  (Savici *et al.*, 2010 ▸) proposed for the Spallation Neutron Source], and the recent development  (Duc *et al.*, 2018 ▸) of a 40 T pulsed-field magnet, with spatial limitations similar to those of the HFM, shows that the subject of experiments in restricted geometry conditions remains relevant to the neutron scattering community. Therefore, in the future the *EXEQ*/*InEXEQ* software may be used as a reference for future instrument design and also adapted to be used for coverage calculations on other instruments.

## Supplementary Material

EXEQ input file corresponding to the Figure 1. DOI: 10.1107/S1600576720011942/in5041sup1.txt

EXEQ input file corresponding to Figure 2. DOI: 10.1107/S1600576720011942/in5041sup2.txt

EXEQ input file corresponding to Figure 3. DOI: 10.1107/S1600576720011942/in5041sup3.txt

EXEQ input file corresponding to Figure 4. DOI: 10.1107/S1600576720011942/in5041sup4.txt

InEXEQ input file corresponding to Figure 5. DOI: 10.1107/S1600576720011942/in5041sup5.txt

InEXEQ input file corresponding to Figure 6. DOI: 10.1107/S1600576720011942/in5041sup6.txt

InEXEQ input file corresponding to Figure 7. DOI: 10.1107/S1600576720011942/in5041sup7.txt
